# Sublingual indocyanine green films for non-invasive swallowing assessment and inflammation detection through NIR/SWIR optical imaging

**DOI:** 10.1038/s41598-020-71054-2

**Published:** 2020-08-19

**Authors:** André O’Reilly Beringhs, Surya Pratap Singh, Tulio Alberto Valdez, Xiuling Lu

**Affiliations:** 1 Department of Pharmaceutical Sciences, School of Pharmacy, University of Connecticut, Storrs, CT 06269 USA; 2 School of Medicine, Otolaryngology – Head and Neck Surgery, Stanford University, Palo Alto, CA 94305 USA; 3 Present Address: Department of Biosciences and Bioengineering, Indian Institute of Technology Dharwad, Dharwad, Karnataka 580011 India

**Keywords:** Biomedical materials, Molecular imaging, Rheumatic diseases

## Abstract

Indocyanine green (ICG) is the most commonly used FDA-approved agent for clinical optical imaging, administered through injections only, due to its poor membrane permeability. Although ICG has vast potential for non-invasive non-radioactive imaging in patients, the clinical applications are limited by the invasive administration and short half-life in blood circulation. To expand the clinical value of ICG, non-toxic chitosan-based ICG-loaded films were designed for sublingual administration for near-infrared (NIR) and short-wave infrared (SWIR) optical imaging. Two film formulations were developed with different ICG release rates. Mold-casted self-emulsifying films rapidly released ICG (80% in 4 h) in the form of nanosized droplets, which were mostly swallowed and produced significant contrast of upper digestive tract to enable in vivo swallowing evaluations using NIR/SWIR imaging. Regular films released ICG slowly (80% in 25 h), allowing for steady absorption of ICG to systemic circulation. Inflammation in mouse feet was detected within 30 min after sublingual administration with a 1.43-fold fluorescence increase within 1 h at the inflammation sites, comparable to a 1.76-fold increase through intravenous injection. Administering ICG using sublingual films displayed notable potential for non-invasive diagnosis and monitoring of inflammatory conditions and swallowing disorders, addressing a current need for alternatives to ICG parenteral administration.

## Introduction

Fluorescence imaging within the near infrared window (NIR, 700–1,000 nm) has enabled new technologies for preclinical and clinical applications^1^. Compared with conventional diagnostic imaging techniques (e.g. X-ray computed tomography, positron emission tomography, magnetic resonance imaging), NIR imaging provides low cost and allows for real-time high-sensitivity molecular imaging, without ionizing radiation^2^. A variety of NIR fluorophores are commercially available, including indocyanine green (ICG) which has been approved by the U.S. Food and Drug Administration (FDA) for human clinical use in angiography, blood flow evaluation and hepatic function assessment^3,4^. ICG is remarkably promising as an optical imaging contrast as it can also be detected under short-wave infrared wavelengths (SWIR, 1,000–2000 nm)^5,6^, conferring significant improvement in imaging sensitivity and light penetration depth, while substantially reducing tissue autofluorescence^5,7^.

The administration of ICG in the clinical setting is currently performed either intravenously (i.v.) or intradermally, requiring invasive procedures which limits utilization in sensitive populations, such as pediatric patients^8–10^. Furthermore, this approach requires immediate imaging post contrast administration or multiple contrast dosing for monitoring due to the rapid clearance of ICG in systemic circulation^11^. Yet, there is an absence of formulations on the market that allow for the administration of ICG in a more convenient and patient-friendly manner, without need for multiple and invasive administrations. The choice of route of administration in this case is limited by the biopharmaceutical characteristics of ICG. The oral route, non-invasive and usually preferred from a patient compliance perspective, cannot be reasonably considered for ICG administration due to its perceived poor oral bioavailability^12^, directly associated with the dye’s low membrane permeability, gastric instability, first-pass hepatic metabolism and fast excretion from liver to bile^11,13^. When administered orally, ICG is absorbed in the digestive system, accessing the hepatic portal system and being carried to the liver via portal vein before it reaches systemic circulation. Molecules displaying high liver extraction rates such as ICG (hepatic extraction rate in healthy humans ≈ 70%^14,15^) have their blood concentrations greatly reduced prior to reaching systemic circulation, and therefore greatly reducing bioavailability. In this sense, the sublingual route has many advantages when compared with oral that can assist increasing systemic exposure of ICG: (1) Sublingual rate of absorption is higher due to thinner membrane thickness^16,17^, allowing for easier and faster mass transfer; (2) low degree of keratinization^17^, facilitating permeability; and most-importantly (3) molecules absorbed from the buccal cavity have direct access to the systemic circulation via jugular vein, by-passing first-pass hepatic metabolism associated with oral absorption and increasing bioavailability^18,19^.

In order to address the current gap in novel technologies for patient-friendly administration of ICG, our group developed ICG film formulations for sublingual administration with mucoadhesive properties^20^. We hypothesized sublingual films could promote enhanced systemic exposure of ICG by promoting sublingual absorption while steadily releasing the dye in a sustained manner. Furthermore, due to the intended sustained release capabilities of these sublingual films, their potential use for optical imaging-guided dysphagia assessment was also explored as excess dye (not absorbed from the sublingual space) would likely promote optical contrast within the upper gastrointestinal tract as it is swallowed with saliva.

Considering ICG displays high aqueous solubility^3^, absorption is mainly limited by the absorption rate rather than dye dissolution. In that sense, an immediate release oral dosage form (e.g. conventional tablet) would not promote adequate systemic delivery of ICG as the entire dose is available for absorption at once but kinetically limited by slow absorption of ICG crossing the gastrointestinal wall into the blood circulation^21,22^. Sublingual films containing ICG may foster systemic exposure by steadily releasing smaller doses of ICG into the sublingual vasculature over a prolonged period of time, partially bypassing first-pass metabolism with direct absorption. Portion of the released ICG dose may be swallowed, allowing for diagnosing dysphagia and other esophageal disorders via optical imaging. Systemically absorbed ICG will facilitate constant monitoring of the vasculature and can be used to monitor inflammation and disease progression due to the prolonged release of the film formulations. Chitosan-based mucoadhesive films were selected as platform of choice due to flexibility of use, easy of application and potential of being employed for controlled release purposes. Chitosan has been selected as film-forming mucoadhesive polymer for this platform as it has been reported to be non-toxic for oral applications^23^ and it is capable of fostering mucoadhesion via electrostatic interactions with negatively charged mucin proteins present in mucus^23–25^.

## Results

### Self-emulsifying concentrate design

A Quality by Design (QbD) approach was taken to investigate the impact of critical SEDDS components on droplet size and concentration. SEDDS components and concentration ranges were determined based on previous studies conducted by our group^26^. A mathematical model was developed by means of a Response Surface Methodology design to describe changes in responses as a function of the composition of the microemulsion. The final experimental matrix, with the respective factors, levels and responses found, is provided in Table 1.

**Table 1:** Experimental domain of the Response Surface Methodology IV-Optimal design and responses obtained for each run.

Run	Factors					Mean droplet size (nm)	Droplets/mL
	(A) Castor Oil (%)	(B) Tween 80 (%)	(C) Kolliphor RH 40 (%)	(D) PEG 400 (%)	Water (%)		
1	20.0	9.6	1.0	11.7	57.7	154.1	3.97 × 10^9^
2	19.0	11.1	34.0	18.5	17.4	136.5	2.20 × 10^9^
3	6.9	16.0	34.0	2.5	40.7	118.1	1.14 × 10^9^
4	20.0	16.0	34.0	1.0	29.0	111.5	1.03 × 10^9^
5	20.0	9.6	1.0	11.7	57.7	145.4	3.27 × 10^9^
6	12.4	16.0	1.0	1.0	69.6	149.3	3.36 × 10^9^
7	10.5	8.5	17.5	15.5	48.0	124.4	9.60 × 10^8^
8	15.7	1.0	1.0	30.0	52.3	156.4	3.12 × 10^9^
9	20.0	1.0	34.0	22.8	22.2	109.6	3.24 × 10^9^
10	1.0	16.0	34.0	18.7	30.3	117.3	9.95 × 10^7^
11	20.0	15.3	18.0	30.0	16.8	146.6	5.39 × 10^9^
12	11.1	16.0	18.3	15.6	39.0	121.6	9.75 × 10^9^
13	20.0	2.1	34.0	5.4	38.5	118.5	1.82 × 10^9^
14	1.0	1.0	34.0	6.8	57.2	123.5	2.10 × 10^8^
15	20.0	1.0	15.2	1.0	62.8	137.0	4.50 × 10^9^
16	2.0	16.0	1.0	30.0	51.0	129.5	2.00 × 10^9^
17	13.2	11.0	1.0	28.3	46.6	137.7	1.42 × 10^9^
18	1.0	12.4	17.3	1.0	68.3	134.6	2.47 × 10^8^
19	10.5	8.5	17.5	15.5	48.0	122.9	1.66 × 10^9^
20	8.1	9.3	34.0	30.0	18.5	129.6	1.39 × 10^8^
21	1.0	1.0	19.5	30.0	48.5	123.3	1.23 × 10^8^
22	8.1	9.3	34.0	30.0	18.5	132.3	1.64 × 10^8^
23	1.0	12.4	17.3	1.0	68.3	128.5	3.80 × 10^8^
24	4.9	1.0	1.0	1.0	92.1	140.5	2.30 × 10^9^
25	10.8	6.7	17.5	15.8	49.2	122.8	1.82 × 10^9^
26	1.0	7.0	1.0	16.5	74.5	130.2	3.06 × 10^7^
27	20.0	5.8	17.4	29.3	27.5	139.2	5.97 × 10^9^
28	11.1	16.0	18.3	15.6	39.0	129.0	1.36 × 10^9^
29	10.8	6.7	17.5	15.8	49.2	125.3	1.66 × 10^9^
30	11.0	7.0	34.0	1.0	47.0	128.2	2.03 × 10^8^

The mathematical model describing the mean droplet size was found significant (*p* = 0.0006), whereas two main factors showed relevant influence: (A) castor oil (*p* = 0.0481) and (C) Kolliphor RH 40 (*p* < 0.0001); following a model described as:

$$Mean\, droplet \,size \,\left(nm\right)= +131.27+4.10*A-0.038*B-10.11*C+1.05*D$$

**Figure 1:**
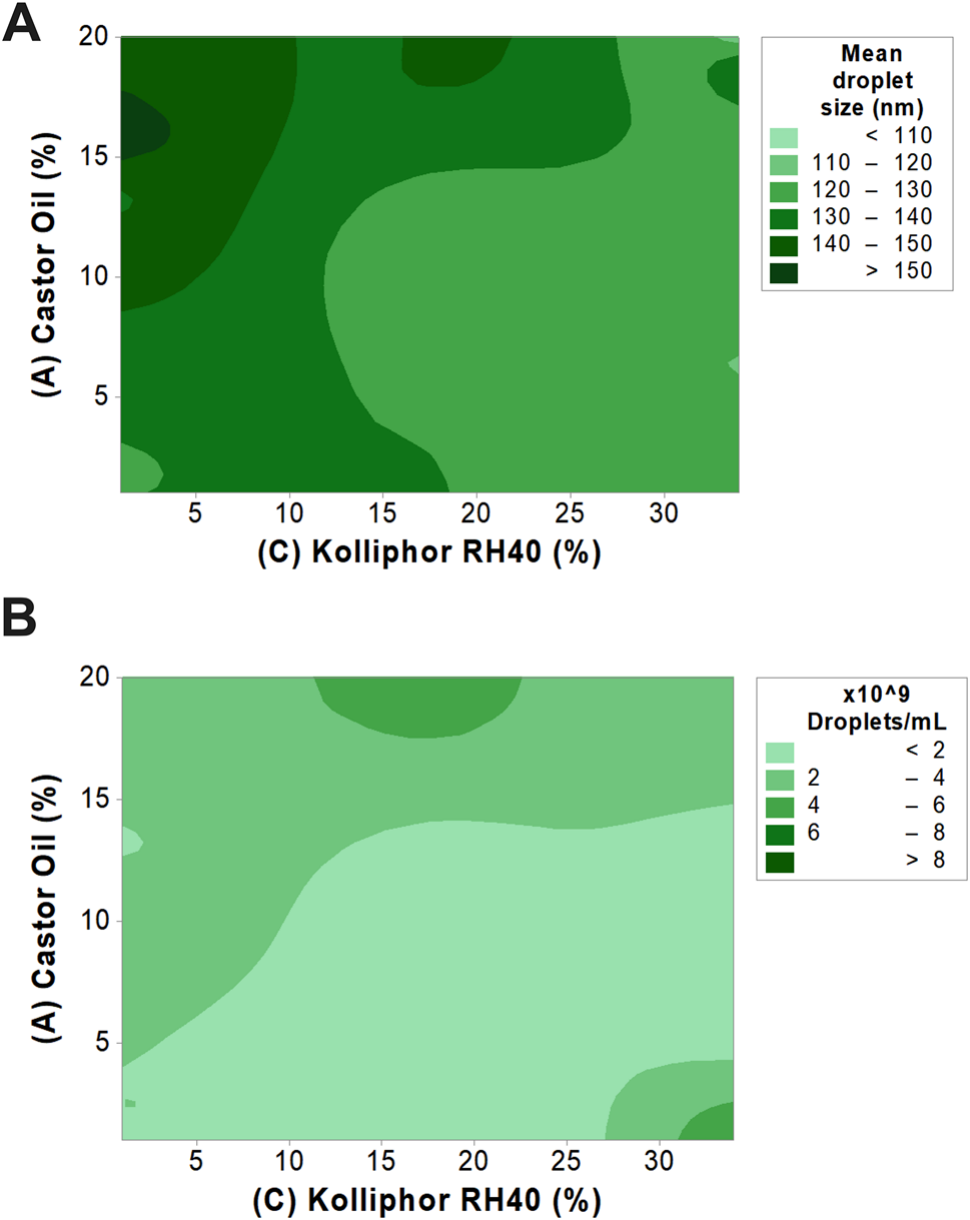
Quality-by-Design assessment of self-emulsifying drug delivery systems via RSM IV-Optimal design. (**A**) Contour plot correlating castor oil and Kolliphor RH 40 contents to mean droplet size of microemulsions. (**B**) Contour plot correlating castor oil and Kolliphor RH 40 contents to microemulsion droplet concentration.

In general, it was observed that the increase in castor oil concentration led to an increase in droplet size as this component acts as the oil filler of the microemulsion (Fig. 1A). An increase in concentration of Kolliphor RH 40 could be associated with an overall decrease of the droplet size (Fig. 1A). Among both factors, Kolliphor RH 40 shows strong influence on the droplet size, as demonstrated by its higher coefficient of − 10.11 versus + 4.10 for castor oil.

The mathematical model describing the average droplet concentration in the microemulsions was also significant (*p* < 0.0001) and the only factor that significantly influenced this response was (A) castor oil (*p* < 0.0001); following a model described as:

$$Droplet \,concentration \,\left({mL}^{-1}\right)= +\,1.796\,\times\, {10}^{9}\,+\,1.571\,\times \,{10}^{9}\,*\,A\,\,-\,\,1.07\,\times \,{10}^{8}\,*\,B-7.53\, \times \,{10}^{8}\,*\,C\,+\,1.673 \,\times \,{10}^{8}*D$$

The influence of castor oil content on the droplet concentration was significantly larger than the other factors, as shown by the equation coefficients. As observed in Fig. 1B, the increase in castor oil increases the number of droplets due to its function as an oily filler.

### Characterization of regular and self-emulsifying films

Based on the RSM assessment, SEDDS composition was standardized as castor oil, Tween 80, Kolliphor RH 40 and PEG 400 at a 20:16:34:30 weight ratio, maximizing droplet concentration and reducing droplet size. SEDDS liquid concentrate was prepared and loaded with ICG. Upon dispersion in simulated saliva (0.5% w/w), the liquid concentrate spontaneously emulsifies forming nano-sized droplets in the form of a microemulsion^26^, yielding a turbid dispersion. The average hydrodynamic diameter and droplet concentration were found as 160.5 ± 2.1 nm and 3.54 × 10^9^ ± 3.87 × 10^8^ droplets/mL, respectively. Upon dispersion, load and encapsulation efficiency were found as 147.2 ± 2.9 µg/mL and 87.3 ± 5.1%.

**Figure 2:**
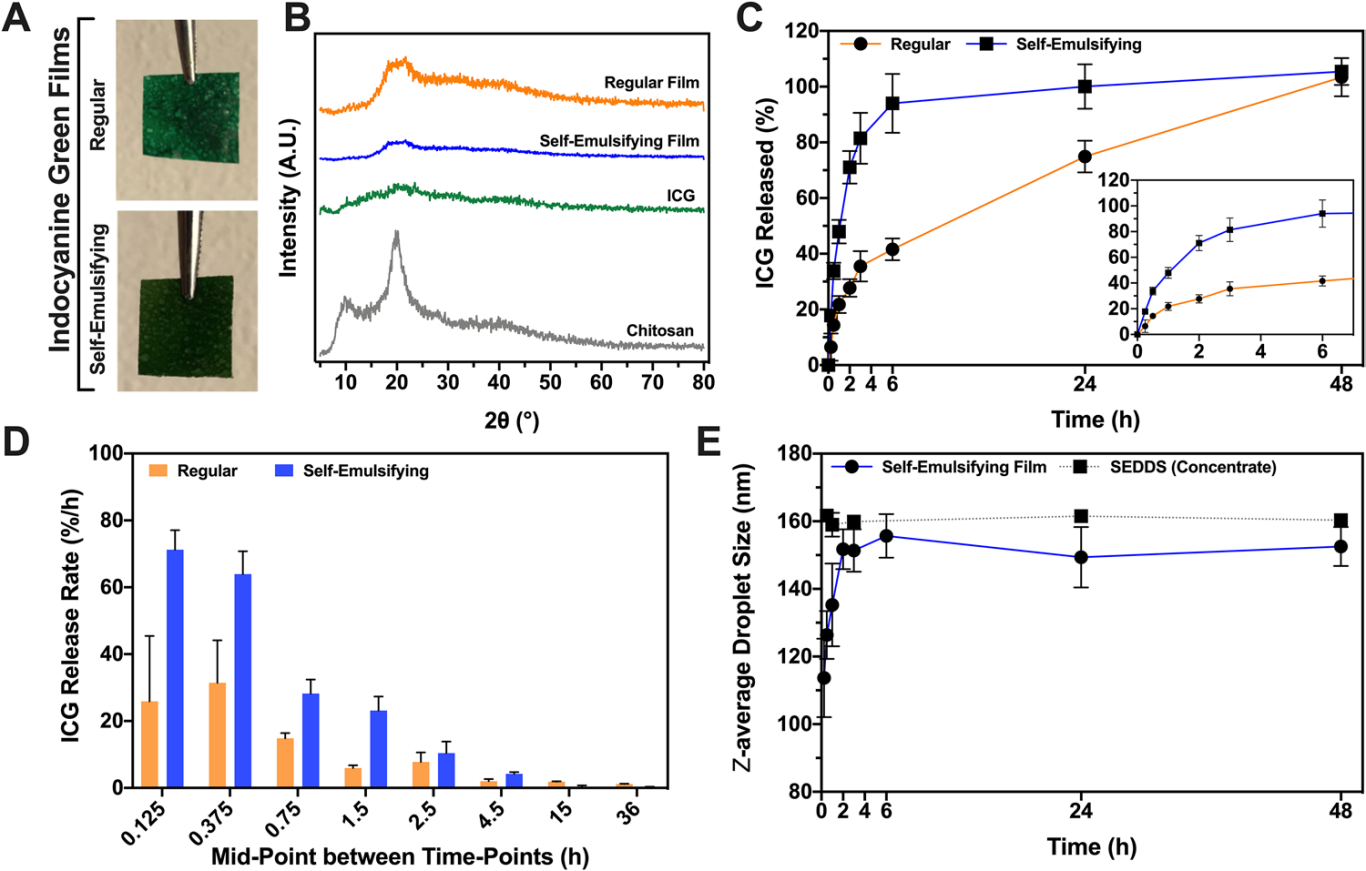
Physical characterization and in vitro performance assessment of ICG-loaded sublingual films. (**A**) Photographs of dried films post cast-molding manufacturing. (**B**) X-ray diffraction patterns for chitosan, ICG and regular and self-emulsifying films. (**C**) In vitro ICG release profiles of regular and self-emulsifying films. Insert depicts initial time-points highlighting differences between profiles. (**D**) ICG release rate over time. (**E**) Dynamic changes in *z*-average microemulsion droplet size over time during release studies.

Under predetermined conditions, regular and self-emulsifying films could be successfully prepared and yielded a dry film-like product, as shown in Fig. 2A. The regular film was constituted solely of chitosan and ICG, whereas the self-emulsifying film contained also the dried self-emulsifying concentrate. X-ray diffraction studies demonstrated the semi-crystalline properties of the chitosan raw material used in film manufacturing (Fig. 2B), showing broad diffractions between 7.5 to 12.5°, and 18.0 to 24.0°. The ICG raw material used was amorphous in nature. Regular and self-emulsifying films were partially amorphous post drying. Characteristic diffractions from chitosan were observed in regular films, and self-emulsifying films at a lower intensity, likely due to the presence of self-emulsifying excipients. Thermogravimetric analyses revealed differences in residual moisture post drying (*p* = 0.0097), with regular and self-emulsifying films showing average moisture contents of 2.0 ± 0.5% and 4.2 ± 0.6%, respectively. Moisture content values were used for dosing corrections in further studies.

### In vitro release studies and microemulsion assessment

Films were subjected to in vitro release studies in simulated saliva as means to characterize the performance of these products in a comparative manner. The release performance comparison showed significant differences between tested products (Fig. 2C; Similarity factor, *f*_*2*_ = 21.4). In general, regular films released ICG at a slower rate when compared with self-emulsifying films. Self-emulsifying films showed a pronounced burst release, which was moderate for the regular film. These findings are corroborated by the observed changes in release rate (Fig. 2D) comparing initial with later time-points. This performance difference is intended by design as the self-emulsifying film releases ICG faster in the form of microemulsion droplets, whereas the regular film relies solely on the hydration of chitosan and dissolution of ICG for release to occur, a much slower process in essence.

Interestingly, release samples collected during testing of the self-emulsifying films showed deviation in average microemulsion droplet size when compared with the droplet sizes obtained for the control SEDDS concentrate (Fig. 2E). At earlier time-points, droplet sizes ranged from 110 to 140 nm, trending upwards over time. At the last time-point of the study, the average droplet size was 152.5 ± 5.8 nm, which is smaller than the expected droplet size for this microemulsion (control, 160.5 ± 2.1 nm). Also, the final droplet concentration was found to be significantly lower than expected (1.05 × 10^9^ ± 0.57 × 10^8^ droplets/mL for release samples *versus* 3.54 × 10^9^ ± 3.87 × 10^8^ droplets/mL for control SEDDS post full reconstitution). These findings suggest microemulsions formed from self-emulsifying films did not display the same composition as full microemulsions derived directly from the control SEDDS concentrate.

From the solid film matrix, not only ICG has to be released but also the distinct components of the microemulsion (i.e. castor oil, Tween 80, Kolliphor RH 40 and PEG 400). The physical characteristics of formed microemulsions are directly dependent on an interplay among the aforementioned components, therefore indicating a change in composition ratio during release from self-emulsifying films. The data collected through the RSM design (Fig. 1) indicated a reduction in droplet size and concentration, as observed during release studies, can be associated with a retention of castor oil within the film matrix, and an increase on the ratio of Kolliphor RH 40 released to the aqueous medium.

### Sublingual film administration for swallowing evaluation

**Figure 3:**
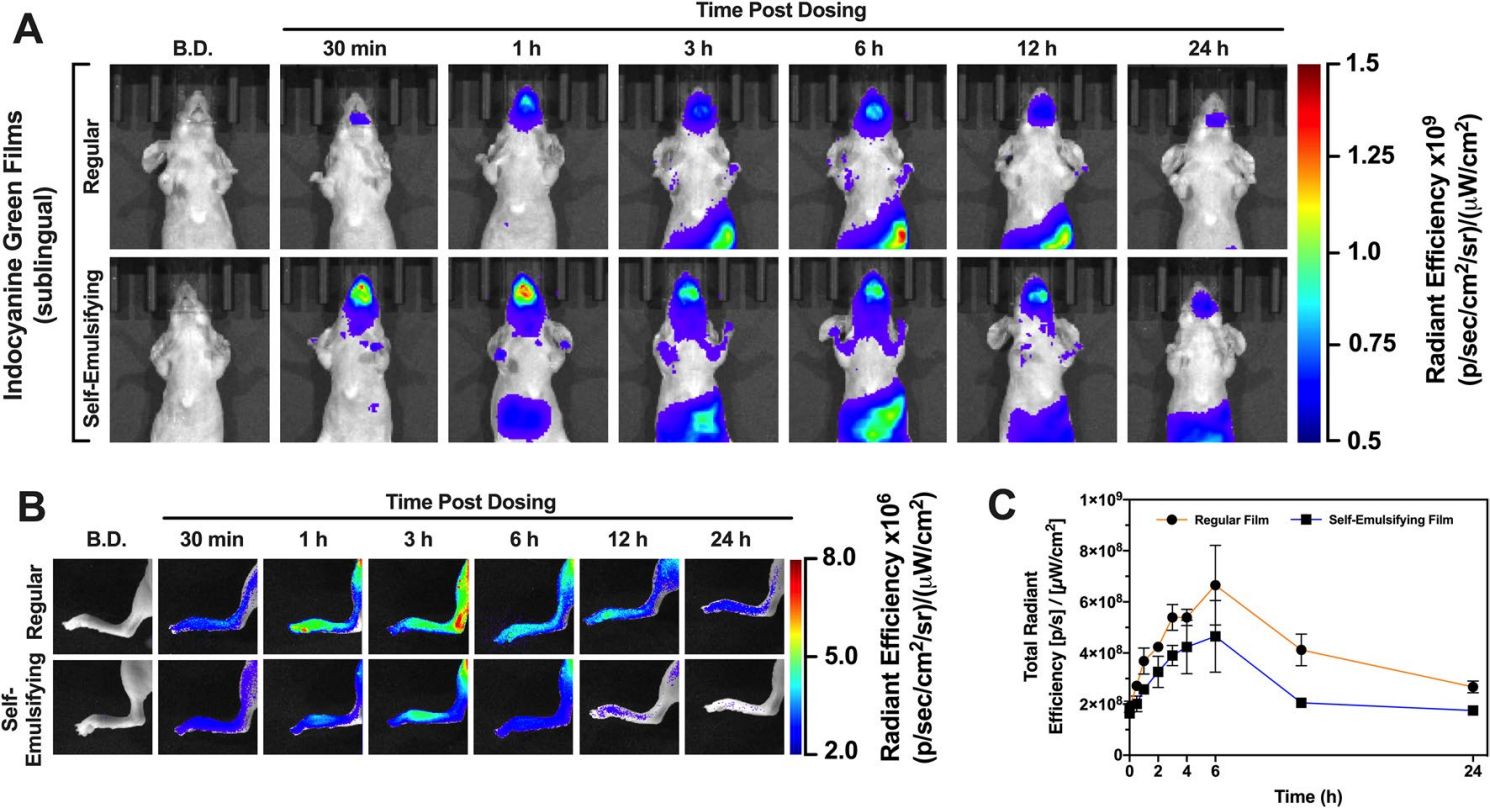
(**A**) Representative in vivo optical imaging of mice upper body upon administration of regular and self-emulsifying sublingual films for swallow evaluation applications over time. (**B**) Representative in vivo optical imaging under narrow field of view of left back paw indicating presence of ICG in blood circulation post sublingual administration of regular and self-emulsifying films. (**C**) Total radiant efficiency quantification of left back paw signal indicating systemic exposure to ICG over time.

Films intended for upper gastrointestinal tract imaging (swallowing evaluation) were administered to the sublingual compartment of mice using forceps. Figure 3A displays representative images comprised of the superposition of photographs and fluorescence signal. The field of view was adjusted to highlight the upper gastrointestinal tract and investigate the effect of film type (regular *versus* self-emulsifying) on the fluorescence signal in the region of interested.

In general, the self-emulsifying film showed promise in its use for swallowing evaluation when compared with the regular film, as showcased by the optical imaging data (Fig. 3A). As early as 30 min post dosing, intense fluorescence signal could be detected from the mouth, where the film is adhered to, down the animal’s throat. The upper gastrointestinal tract signal is sustained for up to 6 h and reducing afterwards as result of depletion of ICG for the sublingual film reservoir. These findings are consistent with the in vitro release results (Fig. 1C) which demonstrated the fast release properties of the self-emulsifying film.

The regular films displayed a modest performance with regards to swallow evaluation (Fig. 3A). This film does not contain self-emulsifying excipients and thus relies solely on polymer hydration and dye dissolution for release to occur. As result, the release kinetics is slower when compared with the self-emulsifying film (Fig. 1C), corroborating the in vivo performance observed for this application.

### In vivo systemic delivery of ICG post sublingual dosing

NIR optical imaging was performed to evaluate the systemic delivery of ICG post administration of ICG-loaded sublingual films. A narrow field of view was used for imaging in order to highlight the fluorescence signal from the animals’ back paws. Paws are highly vascularized and show low tissue autofluorescence. Therefore, fluctuations in fluorescence signal can be correlated with fluctuations of ICG concentration in blood circulation. This approach allows for the estimation of systemic delivery of fluorescent compounds without use of invasive experimental procedures.

Optical imaging data is shown in Fig. 3B and the quantification as a function of radiant efficiency is provided in Fig. 3C. It can be ascertained that regular films promoted an overall higher systemic absorption of ICG compared with the self-emulsifying films. Both formulations led to a similar kinetic behavior with gradual increase in fluorescence signal while the rate of absorption of ICG is higher than its rate of elimination. Fluorescence signal reaches a maximum between 4 and 5 h, followed by decrease in signal as ICG in the films start to get depleted, thus reducing its rate of absorption and leading to a reduction in concentration in blood.

The self-emulsifying film, although capable of releasing higher doses at faster rates, does not promote improved systemic delivery of ICG when compared with the regular film, likely due to swallowing of the dye and partition requirements among microemulsion droplets (where ICG is dissolved), saliva and sublingual membrane. Bioavailability of ICG from an absorption site is not limited by dissolution rate but rather by the molecule’s permeation rate through membranes and first-pass metabolism when applicable. The regular film released free ICG at a slower rate, but it was capable of sustaining the release for a longer period of time, as corroborated by the in vitro release study results (Fig. 1C). This allows for both sublingual and gastrointestinal absorption over time. Self-emulsifying film released ICG loaded in nano-sized droplets (microemulsion form, not free in solution), possibly creating partition issues between the droplets’ lipidic core and gastrointestinal tract membranes. In this sense, a comparatively higher systemic exposure over time can be achieved using the regular film, whereas the self-emulsifying film led to a higher gastrointestinal ICG content.

### Sublingual dosing of ICG-loaded films for inflammation detection via NIR optical imaging

**Figure 4:**
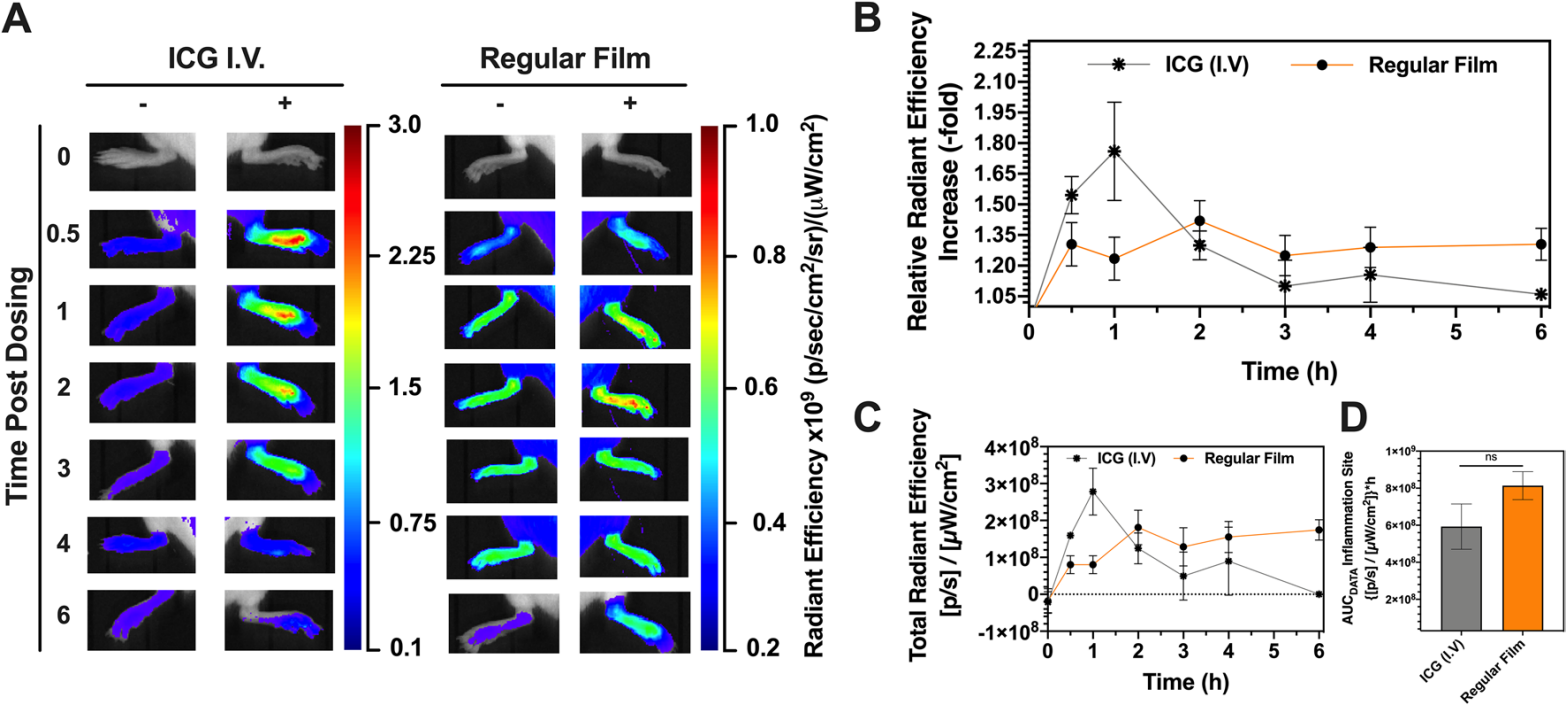
Inflammation detection via in vivo NIR-imaging post ICG-loaded film sublingual dosing. (**A**) In vivo optical imaging of mice under narrow field of view post inflammation induction. “–“ refers to control paw, which received saline i.pl. “ + ” refers to inflamed paw, which received LPS i.pl. (**B**) Relative radiant efficiency fold increase when comparing inflamed paw with control paw. (**C**) Total radiant efficiency quantification of inflamed site over time. (**D**) Exposure of inflamed site to fluorescent reporter as represented by the Area Under the Curve (AUC_DATA_) extrapolated from dataset C.

To assess the potential of using sublingual films for inflammation imaging, the regular film platform was employed as it demonstrated higher overall systemic delivery when compared with the self-emulsifying one. Back paw imaging was conducted and comparisons between non-inflamed paw (saline i.pl.; left paw) and inflamed paw (LPS i.pl.; right paw) were performed (Fig. 4A) in each animal. Animals were treated with either sublingual regular film or ICG administered intravenously as control. Results were plotted as a function of relative radiant efficiency increase when compared inflamed and non-inflamed paws (Fig. 4B), thus minimizing the interference of systemic ICG and highlighting ICG extravasation into the inflammation site.

The highest fold increases in radiant efficiencies were 1.76 ± 0.24-fold increase for ICG I.V. *versus* 1.43 ± 0.10-fold increase for regular film. The ICG i.v. group reached maximum fold-increase in signal at the inflammation site within 1 h post administration, significantly reducing its signal after this time-point. These findings are consistent with the pharmacokinetics of ICG administered intravenously, being rapidly removed from blood circulation^13^. The regular film, on the other hand, promoted a significant fold-increase in fluorescence at the inflammation site in a sustained manner. Instead of increasing the signal exponentially for a short duration of time, as observed for ICG intravenously administered, the fold-increase was maintained for a much longer period of time. In fact, a significant fold-increase in fluorescence was observed for up to six hours, the duration of the experiment. Kinetic differences between the two groups are due to pharmacokinetic differences between the two routes of administration: I.V. group reaches maximum fold-increase at early time-points as the full dose is immediately available on blood circulation, and dynamic changes in signal are governed by ICG’s extraction from blood circulation by liver. ICG sublingual film—on the other hand—promoted a moderate fold increase in signal when compared with intravenous route as only a fraction of the full dose is bioavailable at any given time, and dynamic changes in signal are governed by a balance between ICG’s absorption and elimination over time.

The signal contribution of ICG in the inflamed paw could be estimated by subtracting the radiant efficiency of the healthy paw from its signal (Fig. 4C). Following the trapezoidal rule, AUC_DATA_ values were estimated as a representation of site exposure. The AUC_DATA_ quantified at the inflammation site showed no significant differences between groups that received ICG intravenously or regular films sublingually (*p* > 0.05; Fig. 4D). This demonstrated the regular film promoted similar exposure of the inflammation site to ICG over time when compared with intravenous administration.

### *Whole-body* SWIR imaging and inflammation detection

**Figure 5:**
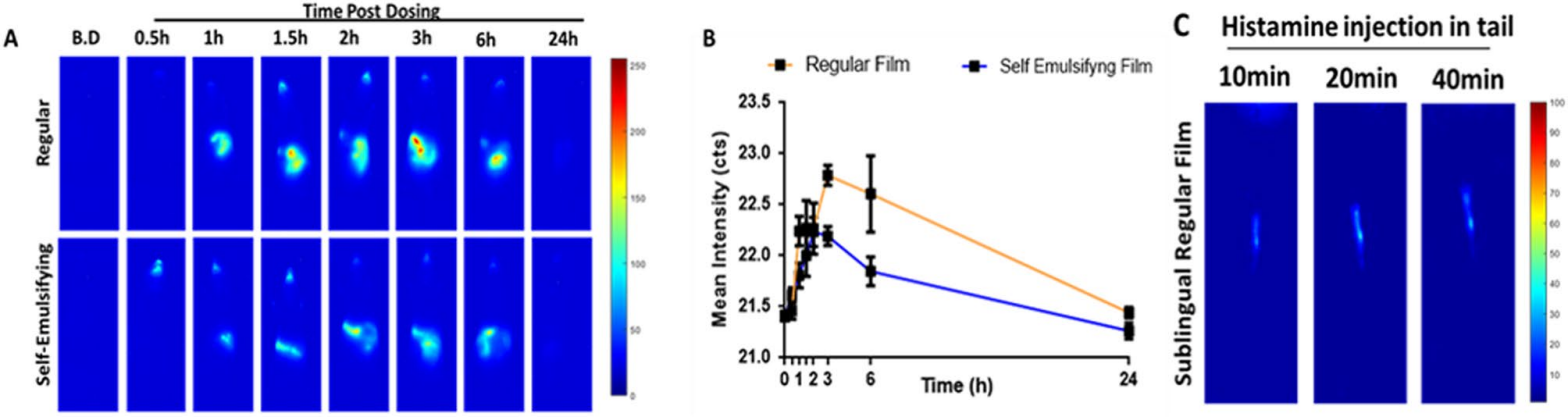
In vivo SWIR whole-body imaging and inflammation detection post ICG-loaded film sublingual dosing. (**A**) Representative in vivo whole-body SWIR images indicating presence of ICG in the blood circulation post sublingual administration of regular and self-emulsifying films. (B.D.-before dosing) (**B**) Mean intensity count of the total signals in the image indicating systemic delivery of sublingual ICG over time. Data is presented as mean ± standard deviation. (**C**) In vivo SWIR imaging of mice tail for evaluating extravasation of ICG post inflammation induction. Inflammation was induced using intramuscular injection of histamine in tail after placing regular sublingual ICG film for 60 min.

SWIR imaging can provide greater contrast and resolution in biological tissues due to decreased scattering. To evaluate the efficacy of SWIR imaging in identifying the systemic distribution of sublingual ICG films whole body imaging using 793 nm excitation and 1,000 nm longpass filters was performed using an in-house SWIR setup. Corroborating with the findings of the optical imaging, similar ICG in vivo kinetics across regular and self-emulsifying films were observed (Figs. 5A,B). Strong signal originating from the mouth can be seen as early as 30 min post dosing with self-emulsifying films and the signals can easily sustain up to 6 h (Fig. 5B). Due to high sensitivity of SWIR imaging, significant intensity changes could be observed as early as 30 min post dosing. Reduction of mouth signals in mice over time can be due to head movement during the imaging and partial ICG depletion from dye reservoir. Continuous decrease of the mouth signal indicates further depletion of the dye reservoir from mouth, caused by its direct absorption from the sublingual compartment as well as release into saliva and further dye ingestion. Due to absence of any self-emulsifying excipients, the release kinetics of the regular films were relatively slower when compared with self-emulsifying ones. However, overall systemic delivery was qualitatively higher for the regular films, corroborating previous observations from the in vivo NIR optical imaging study.

Using a histamine-induced tail inflammation model, the efficacy of regular films in identifying inflammation sites through SWIR imaging was investigated (Fig. 5C). Regular films were used due to their ability to promote higher ICG systemic delivery when compared with self-emulsifying films. Histamine, a vasodilator agent, was injected into the subcutaneous space of the tail to induce inflammation. Dynamic changes of fluorescence signals within 10 min after histamine injection revealed high sensitivity of ICG films in tracking inflammation (Fig. 5C). Overall findings of SWIR imaging are supportive of the potential applicability of these films for continuous long-term monitoring.

## Discussion

ICG has been safely employed in medical imaging for over five decades, but only recently its use for fluorescence imaging has significantly increased concurrent with advances in high resolution camera and video technologies. Medical and surgical applications of ICG range from but not limited to lymph node identification, hepatic function assessment, vascular angiography and detection of tumors and inflammation^3,4^. Although being considered as a fluorescent reporter for a wide variety of applications, not enough focus has been given to the development of novel formulations which can broaden the scope of ICG clinical use, especially focusing on non-invasive routes of administration. In general, during the last decade, a wide-range of nanoparticle-based ICG-loaded formulations have been developed, including but not limited to liposomes^27–32^, micelles^33–35^ and lipid nanoparticles^36^. These formulations are mainly intended for targeted imaging (e.g. tumor targeting) or photosensitization, and usually require invasive administration (i.e. intravenous or subcutaneous route). Our formulation strategy focused on developing sublingual dosage forms to circumvent invasive routes of administration while conferring novel functionalities to ICG in clinical imaging. As far as the authors are aware, there are no significant efforts reported in the literature on the development of ICG formulations for sublingual administration as described herein.

One application in which ICG has the potential of advancing is swallowing evaluation, which currently requires the administration of radiopaque contrasts (i.e. barium sulphate) followed by X-ray fluoroscopy. This approach could be substituted by optical imaging using NIR/SWIR fluorophores such as ICG, circumventing ionizing radiation and limited set up flexibility associated X-ray-based imaging methodologies. This is especially desired in premature infant populations, commonly subjected to dysphagia evaluations, and yet highly sensitive to radiation exposure^37^. Furthermore, recent studies have demonstrated the potential use of ICG not only for NIR imaging but also for imaging under SWIR region (1,000–2000 nm)^5,6^, further advancing detection sensitivity, reducing tissue autofluorescence and increasing signal depth penetration^7^.

Current ICG formulations on the market (e.g. IC-GREEN, sterile lyophilized powder for reconstitution) are intended for parenteral administration mainly due to bioavailability limitations associated with oral ICG administration. This compound displays high aqueous solubility and low membrane permeability, yielding similar characteristics to BCS class 3 drugs^38^. For this category of compounds, oral administration is challenging as the absorbed dose can be kinetically limited by its absorption rate, which is affected by membrane permeability^39^. Furthermore, first-pass hepatic metabolism is a major issue associated with the reduced gastrointestinal bioavailability of ICG^13^. In this sense, sublingual administration is promising as the sublingual space has low membrane thickness and keratinization^18^, potentially fostering systemic absorption when compared to other gastrointestinal membranes, and assisting bypassing first-pass metabolism^40^. Another valid approach to circumvent the aforementioned limitations is employing controlled release systems that are capable of sustaining drug release for a prolonged period of time. Assuming equal doses are administered, an immediate release oral dosage form would be unsuitable for ICG administration as the entire ICG dose is available for absorption immediately but hindered by the absorption rate. Releasing the same total dose over a prolonged period of time likely leads to higher systemic exposure as the kinetic limitation of permeability is reduced when smaller doses are available for absorption at a certain time. Therefore, a slow-release sublingual platform to administer ICG shows promise in overcoming the aforementioned limitations.

Considering the current mode of administration of ICG in the clinic, there is a need supporting the development of novel forms of administering ICG to patients when parenteral route is undesired or unsuited for. To address this issue, we have proposed two distinct platforms to deliver ICG in a non-invasive manner, using biocompatible sublingual films.

The first platform, a chitosan-based ICG-loaded film (regular film), capable of releasing ICG slowly into the sublingual cavity, demonstrated potential increasing systemic exposure of ICG. This platform released the dye steadily and slowly, supplying the sublingual space with small doses of free ICG immediately available for sublingual absorption and further bypassing first-pass metabolism. Unabsorbed doses dissolved in saliva are swallowed and further systemic absorption can occur within the upper and lower gastrointestinal tracts.

Since regular films were able to significantly increase systemic ICG exposure, this platform was tested against a preclinical inflammation model to verify whether it would be suitable for inflammation detection via optical imaging. For this purpose, ICG must be absorbed and become bioavailable in the blood stream to reach the inflammation site of interest. Considering inflammation leads to increase in blood flow, vessel permeability and fluid extravasation^41^, it was expected systemic ICG would also extravasate into the inflammation site. As consequence, an increase in fluorescence signal was expected when comparing an inflamed area with an equivalent non-inflamed one. This effect has been confirmed through in vivo optical imaging, where a significant increase in paw fluorescence signal could be observed in the inflammation model. The response intensity for ICG intravenously administered was higher at early time-points, but rapidly decreased due to ICG clearance from the body. In this case, the full ICG dose was immediately available in the blood stream. The use of regular ICG films led to a lower response intensity, which lasted for a longer period of time due to its sustained release and depot-like properties, addressing the undesired need for multiple ICG intravenous administrations in order to sustain adequate fluorescence signal at the inflammation site.

The second platform, an ICG-loaded SEDDS incorporated into a chitosan-based film (self-emulsifying film), displayed much faster release kinetics and was more suited for swallowing evaluations by coating the upper gastrointestinal tract with the fluorescent dye. Self-emulsifying systems are surfactant-rich anhydrous isotropic concentrates which spontaneously form oil-in-water microemulsions upon mixing with aqueous solutions^26^. Upon administration, chitosan hydrates forming a mucoadhesive hydrated layer in the sublingual space and allowing for release of entrapped components, including ICG and self-emulsifying excipients. ICG is steadily released in the form of nano-sized droplets at a high concentration. Under such conditions, ICG-rich saliva is swallowed, creating a fluorescent track that can be dynamically monitored through optical imaging. This approach allows for swallowing evaluations to be conducted, assisting clinical assessment using non-invasive and non-radioactive imaging. Microemulsions formed when spontaneous emulsification occurred showed deviations in composition, likely due to partial retention of SEDDS components within the film, leading to distinct microemulsion physical characteristics. Nonetheless, these changes did not impair the performance of the self-emulsifying films applied sublingually.

Self-emulsifying films released ICG in the form of a microemulsion, and therefore the dye molecules must partition from the oily dispersed droplets into the aqueous environment and subsequently cross gastrointestinal membranes in order to promote systemic exposure. Based on the experimental evidences, the aforementioned effect does not occur efficiently enough to promote increased systemic exposure when compared with regular ICG films. The concept of using a self-emulsifying film targeted initially at increasing bioavailability of this dye mainly due to its fast-releasing properties. However, our results demonstrated the opposite occurs for this specific system. This happens because ICG absorption is mainly limited by absorption rate rather than dissolution rate, as expected for compounds fitting the BCS class 3 classification. ICG in microemulsions must partition out of the liquid emulsion droplet where it is dissolved (hydrophobic core in nature) and into the sublingual membrane in order to be systemically absorbed. On the other hand, regular ICG films only require ICG dissolution in saliva in order to be able to cross the sublingual membrane. Due to the much faster release rate of the self-emulsifying film, most of the dye load is not absorbed sublingually, but rather swallowed with saliva, and therefore more suited for gastrointestinal imaging where systemic absorption is not relevant. The regular film released free ICG (no self-emulsification) as it is solubilized by saliva at a slower rate, fostering sublingual absorption by comparison with the self-emulsifying formulation. These observations were drawn based on the upper gastrointestinal tract imaging (Fig. 3A), which showed higher degree of dye swallowing for the self-emulsifying film when compared with the regular one, associated with systemic exposure observations via optical imaging (Fig. 3B).

## Conclusions

Advances in optical imaging technologies have opened up new possibilities in non-invasive non-radioactive imaging in the clinic. The current scenario led to a market-need for novel delivery approaches for fluorescent reporters. In our study, we addressed this need by developing and investigating preclinically novel chitosan-based sublingual film platforms. The regular film platform promoted ICG systemic exposure by sustaining the release of ICG sublingually over time and it could be used for inflammation detection without the need of multiple intravenous administrations of ICG. The self-emulsifying film platform can be employed for dysphagia evaluations and upper gastrointestinal imaging by releasing the ICG at a fast release rate and coating the upper gastrointestinal tract. The results strengthen the ability of ICG to be used for NIR/SWIR optical imaging employing patient-friendly, biocompatible and non-invasive contrast delivery platforms.

## Methods

### Materials

Indocyanine green (ICG) was purchased from the United States Pharmacopeia (USP reference standard). Chitosan (medium molecular weight), Kolliphor RH 40 (macrogolglycerol hydroxy stearate), histamine (H7125) and lipopolysaccharides from *Salmonella typhosa* (LPS) were obtained from Sigma-Aldrich (USA). Castor oil was acquired from Aqua Solutions (USA). Poly(ethylene glycol) M.W. 400 (PEG 400) and Tween 80 were acquired from Fisher Scientific (USA). Isoflurane (USP grade, Isothesia) was procured from Henry Schein (USA). Simulated saliva (pH 6.75) was prepared as described by Koland and collaborators^42^. All other reagents and solvents were of analytical grade unless stated otherwise.

### Self-emulsifying drug delivery system (SEDDS) design

A Design of Experiments (DoE) approach was taken to investigate how different formulation parameters could affect droplet size distribution and concentration of formed microemulsions upon SEDDS dispersion. A Response Surface Methodology (RSM) IV-Optimal design was prepared to minimize the integrated prediction variance across the design space, totalizing 30 SEDDS formulations varying the lipid components. The experimental domain comprised of (A) castor oil, 1–20%; (B) Tween 80, 1–16%; (C) Kolliphor RH 40, 1–34%; and (D) PEG 400, 1–30%, based on a previous report^26^. Droplet size distributions and concentrations were determined by dynamic light scattering and nanoparticle tracking analysis as described in section “Microemulsion characterization”. Regression analysis was performed, and the statistical significances of the factors were determined via Analysis of Variance (Design-Expert 7.0, Stat Ease Inc, USA). Contour plots were graphed using Minitab 18 (Minitab LLC., USA).

### Microemulsion characterization

Microemulsions formed upon dispersion of SEDDS concentrate in aqueous media were assessed for their hydrodynamic particle size via dynamic light scattering (Zetasizer Nano-ZS, Malvern Instruments, UK) and droplet concentration via nanoparticle tracking analysis (NS500, Nanosight, UK). ICG loading and encapsulation efficiency were determined following droplet separation (Amicon Ultra Centrifugal Unit, regenerated cellulose, MWCO 30 kDa, Millipore, USA) and measuring the concentration of unloaded (free) ICG in the supernatant (continuous phase). Load was calculated as the concentration of ICG entrapped in the dispersed phase, determined via mass balance from the quantified supernatant. Encapsulation efficiency was determined as the percentage of ICG in the dispersed phase when compared with the initial amount of ICG added to the formulation. To evaluate the characteristics of the original microemulsion, a 0.5% (w/w) dispersion was prepared in simulated saliva (n = 6).

### Regular chitosan-based sublingual film preparation

Sublingual films were prepared following a mold-casting methodology. Chitosan stock solution (1% w/w) was prepared in 1% (v/v) acetic acid aqueous solution via high shear mixing (Unguator e/s, Gako International, Germany) using a standard mixing blade for 9 min at 1,450 rpm. In a 20 mL Unguator jar, ICG (2.5 mg) and chitosan stock solution (10 g) were added and subsequently mixed for 9 min at 1,290 rpm. Formulations were inspected to ensure full dissolution of ICG in the polymeric hydrogel prior to mold-casting. 6 g of the hydrogel were poured onto circular plastic dishes (35 mm diameter, 9.62 cm^2^ usable surface area). Formulations were dried in an air-circulating oven at 40 °C for 24 h, forming a solid, thin and malleable film (ICG dose = 2.4% w/w, or 0.16 mg/cm^2^).

### Self-emulsifying chitosan-based buccal film preparation

SEDDS concentrate was prepared by mixing castor oil, Tween 80, Kolliphor RH 40 and PEG 400 (20:16:34:30 weight ratio) on a heated magnetic stirrer at 50 °C until a translucid product was obtained^26^. ICG was loaded onto the SEDDS concentrate (30 mg/g dose) by stirring until fully dissolved. Loaded SEDDS concentrate was weighed in an equivalent amount to yield 2.5 mg of ICG, mixed with chitosan stock solution (10 g) and subsequently homogenized for 9 min at 1,290 rpm (Unguator e/s, Gako International, Germany). The resulting hydrogel was poured onto circular plastic dishes (35 mm diameter, 9.62 cm^2^ usable surface area) and dried in an air-circulating oven at 40 °C for 24 h to form a solid film (ICG dose = 2.4% w/w, or 0.16 mg/cm^2^).

### Characterization of sublingual films

Preformed films were characterized for their moisture content via thermogravimetric analysis using the Q-500 thermobalance (TA Instruments, USA) at a scan range of 30 to 150 °C and a heating rate of 2 °C/min under a 50 cm^3^/min dynamic nitrogen atmosphere. Moisture content was determined by integrating the weight loss up to 120 °C.

X-ray diffraction studies were conducted using the D2 Phaser diffractometer (Bruker, USA) employing a Cu tube as the radiation source (1.54184 Å). Films were horizontally mounted onto sample holders with epoxy cement. Scans were performed from 5 to 80° (2θ) with 0.01°/step increments.

### In vitro release studies

Release studies were conducted using a USP II apparatus (paddle method; AT Xtend, SOTAX Corp., USA). Briefly, films (100 mg, square shaped of equivalent surface area) were pre-wet for 1 min to allow for superficial hydration, followed by quick attachment directly to the bottom of dissolution mini vessels (SOTAX Corp., USA). Pre-warmed simulated saliva (150 mL, 37 °C) was carefully added to the vessels and stirred at 100 rpm for up to 48 h at 37 °C. Aliquots were withdrawn at 0.25, 0.5, 1, 2, 3, 6, 24 and 48 h for quantification via UV–Vis (708 nm; UV-1800, Shimadzu, Japan) following an analytical standard curve in simulated saliva (1–20 µg/mL, n = 6). In vitro release samples were also assessed for hydrodynamic particle size and droplet concentration as described in section “Microemulsion characterization”.

### Animals

In vivo studies were conducted using either nude or severe combined immunodeficiency (SCID) mice as specified in each experimental protocol. All animal procedures were performed in accordance with the National Institutes of Health Guide for the Care and Use of Laboratory Animals and were approved by the Institutional Animal Care and Use Committee (IACUC) at the University of Connecticut and Stanford University. Every effort was taken to minimize animal use and distress during the execution of in vivo studies.

### In vivo swallowing assessment via NIR optical imaging

The potential of using sublingual/buccal films for swallowing evaluation through optical imaging was assessed in vivo by administering regular and self-emulsifying films to the sublingual space of nude mice at a 50 µg ICG/mouse dose (n = 3). Animals were anesthetized (1.5 L O_2_/min, 2.0% isoflurane), films were pre-wet in saline and manually attached to the sublingual mucosa. To ensure adequate adherence, animals were maintained under anesthesia for 30 min post film attachment. Optical imaging was conducted using the In Vivo Imaging System (IVIS) SpectrumCT (PerkinElmer, USA) under epifluorescence mode (excitation = 745 nm, emission = 840 nm) with whole-body automatic exposure.

### In vivo systemic ICG delivery monitored via NIR optical imaging

NIR optical imaging was conducted to semi-quantitatively estimate the systemic delivery of ICG following sublingual administration via ICG-loaded chitosan films. Nude mice were anesthetized (1.5 L O_2_/min, 2.0% isoflurane), films were pre-wet in saline and manually attached to the sublingual mucosa followed by 30 min resting period under anesthesia to ensure film attachment. Optical imaging was conducted using the In Vivo Imaging System (IVIS) SpectrumCT (PerkinElmer, USA) under epifluorescence mode (excitation = 745 nm, emission = 840 nm), with automatic exposure, using a narrow field of view for the camera system (FoV C) to collect back paw fluorescence images in real time with reduced whole-body optical interference.

### In vivo inflammation detection via NIR optical imaging

Lipopolysaccharide (LPS)-induced paw inflammation model was established following previous literature reports^43,44^. Briefly, mice were conditioned under anesthesia (1.5 L O_2_/min, 2.0% isoflurane) and placed on prone position to facilitate intraplantar (i.pl.) administration. Using a 30-gauge needle, 20 µL of saline was administered to the left paw as control, and 20 µL of 200 ng LPS/µL stock solution was administered to the right paw to induce acute inflammation. Immediately, treatments were administered to mice (n = 3). Animals were separated in two groups, one receiving 50 µg ICG/mouse intravenously through tail vein injection, and the other receiving 50 µg ICG/mouse administered sublingually in the form of regular film. Mice were maintained under anesthesia for 30 min post film attachment, ensuring adequate adherence. Optical imaging was conducted using the IVIS Lumina II system (PerkinElmer, USA) under fluorescent mode (excitation = 745 nm, emission = 840 nm) with automatic exposure using a narrow field of view (FoV C).

### In vivo short-wave infrared (SWIR) whole-body imaging

A custom-built setup consisting of a fiber (200 µm core) coupled 793 nm laser (20 W, Lumics LU0793D140-D10DH) was used. The laser is launched in an excitation cube by the fiber and reflected through a mirror (Thorlabs BBE1-E03). The reflected light is passed through a positive achromat (Thorlabs AC254-050-B) to an engineered diffuser (Thorlabs ED1-S20-MD) to provide a uniform illumination over the working area. The average excitation flux at the animal was close to 15 mWcm^−2^ (± 3%). The working area was covered with a blackout fabric (Thorlabs BK5). Fluorescence signals were directed to a 4-inch square first-surface silver mirror (Edmund Optics, 84,448) and then to a filter set (Thorlabs, 2 × FELH01000). At the end, the filtered signals were collected using an Allied Vision Goldeye G-032 Cool TEC2 camera with sensor set point at − 30 °C and equipped with a C-mount lens (Navitar, SWIR-35). The assembly was partially enclosed to avoid exposure to unwanted light. The images were acquired using platform independent Vimba SDK (Allied Vision) with a uniform acquisition time of 10 ms. During imaging mice were placed in supine position over pre-defined area under the influence of isoflurane. Inflammation in the tail was induced by intramuscular injection of histamine (0.02 mg/ml). MATLAB programming environment was used for processing and analysis of the acquired images.
